# Waiting in sport: a kinesiological perspective on a universal psychological experience

**DOI:** 10.3389/fpsyg.2026.1628266

**Published:** 2026-06-10

**Authors:** Mingyun Liu, Yifei Wang, Weimin Li, Chuanping Lei, Xuesong Guo

**Affiliations:** College of Sports Sciences, Qufu Normal University, Jining, China

**Keywords:** competition, kinesiology, preparation, psychological experience, spectatorship, sport, waiting

## Abstract

Waiting is a universal experience in sport, marked by distinct cognitive patterns and affective dynamics that carry psychophenomenological significance. This study investigates the psychogenesis of sport across three key dimensions of sport—preparation for participation, competitive engagement, and spectatorship—through the lens of sport behavioral science. The findings reveal three major domains of psychological processing: (1) During preparation for participation, athletes engage in psychological preparation and emotional regulation during waiting periods. Goal orientation exerts neuromodulatory effects, linguistic self-regulation enhances neural plasticity, and neural motor coding supports a stable cognitive state of readiness. Emotional regulation draws on autonomic neuromodulation strategies, models of directed cognitive resource allocation, and mechanisms of social neuroendocrine regulation. (2) During competition, athletes experience waiting through psychological strategising and stress coping. Tactical neuromodulation under pressure, neuro-metabolic resilience mechanisms, and cognitive restructuring models inform strategising. Stress coping involves neural appraisal reconstruction, sympathetic-vagal balance paradigms, and coordinated social neuroendocrine responses. (3) Concerning spectatorship, spectators’ waiting experiences encompass emotional engagement and psychological resonance. Anticipatory motivation aligns with neural coding theories, empathic stress maps onto neuro-resonance models, and emotional regulation follows neuro-metabolic balancing processes. Therefore, psychological resonance emerges through neurochemical models of group identity, neural embodiment simulations, and co-activation within the social brain network.

## 1 Introduction

The word “wait” derives from the Middle English and Old Northern French term “waitier,” whose early meanings were associated with vigilance, observation, and staying alert. The Oxford Dictionary also lists “lie in wait (for),” “observe closely,” and “remain alert” as related definitions. In modern English, “wait” more often refers to letting time pass before something happens, remaining still, or refraining from action for the time being. Based on this, this paper does not presuppose “waiting” as a negative emotion or negative cognition, but rather understands it as a transitional state characterized by a temporary suspension of action, an undecided outcome, or a next step that has not yet arrived. It may be accompanied by anticipation, focus, and preparation, or by anxiety, tension, and a sense of uncertainty; the specific psychological experience depends on the nature of the situation, task requirements, and individual differences. As a ubiquitous temporal experience and social phenomenon, waiting exists not only in daily life but is also embedded within the highly contextualized practice of sports (Schwartz, 1975; Deng, 2024). In sports, waiting is not a marginal phenomenon but permeates multiple phases, including warm-up preparations, pre-match waiting, game intervals, transitions between rounds, and the spectator experience. Compared to waiting in general life contexts, waiting in sports is more closely embedded in physical preparation, emotional arousal, tactical judgment, and social interaction, and thus often exhibits greater dynamism, situationality, and complexity. However, despite the widespread prevalence of waiting in sports, systematic discussions within the sports science community regarding it as an independent psychological experience remain relatively limited. Existing research has primarily focused on adjacent topics such as pre-competition anxiety, attentional control, emotional regulation, and the experience of suspense, while rarely analyzing “waiting” itself as a phenomenon with a unique psychological structure and contextual significance.

For athletes, waiting is not merely a period of inactivity before a competition begins; it also frequently occurs during pre-competition preparation, breaks between matches, transitions between sets, and before returning to the court. Relevant research has consistently examined athletes’ psychological states in situations of anticipation and uncertainty, with particular attention to issues such as attentional control, anxiety, and emotional regulation (Chadha et al., 2019; Tamminen et al., 2021). For student-athletes, the psychological experience of waiting may also be intertwined with academic demands, scheduling, and role conflicts, presenting a more complex picture. Dual-career research indicates that student-athletes often face both compatibility and conflict between school and sports, and may be at higher risk for stress and burnout (O'Neil et al., 2021; Sorkkila et al., 2020; De Maio et al., 2025). For spectators, waiting is more often characterized by an experience of suspense when the outcome is still uncertain, typically occurring during critical moments, when scores are close, and just before the result is revealed. Research on sports spectatorship also indicates that spectators’ suspense and emotional fluctuations are closely linked to outcome uncertainty (Knobloch-Westerwick et al., 2009). Thus, waiting in sports is not a singular, homogeneous experience but rather one that manifests different psychological connotations and experiential modes depending on the participant’s identity, the specific context, and the demands of the task.

Given these characteristics, this paper does not organize its discussion by demographic group but instead begins with the primary contexts in which sports-related waiting occurs, categorizing them into three dimensions—preparation for participation, competitive confrontation, and spectator experience—for analysis. Specifically, preparation for participation primarily involves psychological readiness and emotional regulation in pre-competition contexts; competitive confrontation primarily involves psychological strategies and stress management during the competition; and spectator experience focuses on the waiting experiences of audiences during moments of uncertainty, high tension, and the period before and after the outcome is revealed. It should be noted that the neurobiological and psychological processes discussed in this paper aim to illustrate the possible mechanisms of waiting in sports, rather than asserting that all individuals will react in the same way. The experience of waiting and its regulatory processes may be influenced by factors such as age, gender, athletic experience, competitive level, sport type, cultural background, spectator identity, situational stress, personality traits, and past experiences of success or failure. Furthermore, the intensity with which the same mechanism manifests may vary significantly across different individuals and scenarios. Based on this premise, the following analysis will adopt a conditional and probabilistic interpretive approach, emphasizing the situational dependence and individual variability of reactions to waiting in sports.

## 2 Waiting in preparation for sport participation—psychological preparation and emotional regulation

### 2.1 Psychological preparation

In sport, participants pursue structured goals—orientation, process, and outcome—which form hierarchical goal systems. These goals activate the dorsolateral prefrontal cortex (dlPFC), establishing a stable cognitive anchor during waiting periods (Locke and Latham, 2013). Research on goal setting based on the SMART principles indicates that goal setting can enhance neural activity associated with attentional control and improve an individual’s allocation of resources to task-relevant information (Berkman et al., 2017). For example, the amplitude of the P300 waveform—which is associated with attentional allocation—increased in the study, typically indicating that individuals were able to focus more intently on the target stimulus during the waiting period. At the same time, anxiety-related overactivity in the amygdala was attenuated, suggesting that goal anchoring may help alleviate the experience of tension during waiting periods (Grupe and Nitschke, 2013). In sports participation, the maintenance of goals during waiting periods may be influenced by relevant neural regulatory processes. Through goal anchoring, this mechanism helps some individuals reduce excessive tension and inattention, thereby playing a role in psychological regulation. Specifically, this type of goal anchoring is commonly observed during the waiting period after warm-ups, brief pauses following registration, and the time before substitute players prepare to enter the game. At these moments, if athletes can stabilize their attention on key movement points, the rhythm of the game, or task execution, the waiting period ceases to be merely a passive waste of time and instead becomes a psychological preparation process prior to formal performance.

In addition to stabilizing attention through goal maintenance, some individuals may also rely on self-talk to engage in linguistic self-regulation during waiting periods. In waiting situations associated with sports participation, self-talk may serve as a form of self-regulation that helps some individuals maintain focus, prepare for action, and stabilize their emotions. Related research suggests that Kross et al. (2014) posited that process-focused self-talk may be associated with processes related to cognitive control and emotional regulation. Furthermore, studies on individual differences in emotional processing indicate that there may be genetic susceptibility differences in the performance of prefrontal-limbic system regulation during stress response (Hariri et al., 2002). However, these findings primarily stem from general experimental tasks or non-sports contexts; therefore, they serve best as indirect support for understanding the role of self-talk during sports waiting periods rather than directly indicating that all participants will benefit in the same way. Actual effects may also be influenced by factors such as age, athletic experience, competitive level, baseline anxiety levels, and self-talk habits within specific cultural contexts. For example, this is particularly common before the starting gun, before a free throw, before a serve, and when substitute players are waiting to enter the game. Compared to vague self-encouragement, brief, clear, and process-oriented prompts—such as “maintain your rhythm,” “stabilize your movements first,” and “execute according to the plan”—are more effective in helping individuals maintain a sense of control and agency during waiting periods.

Mental preparation during waiting periods may also be enhanced through motor imagery. During the waiting phase of sports participation, motor imagery may help some individuals maintain familiarity with movements, technical rhythm, and continuity in their state of readiness. Research on mental rehearsal and motor imagery suggests that imagery training may involve changes in activity in movement-related brain regions and in sensorimotor integration processes, and to some extent shares a representational basis with actual movement preparation (Decety et al., 2021; Holmes and Calmels, 2008). These findings provide theoretical support for understanding self-preparation through mental rehearsal during the waiting phase, though the intensity of this effect may not be consistent across individuals. For athletes with stronger imagery abilities, greater experience in their sport, or more stable technical movements, this approach may be more effective; conversely, for those with limited experience or weaker control over imagery, the effects may be relatively limited. From an applied perspective, short-term motor imagery is particularly suitable for the waiting period immediately before entering the competition venue. By mentally rehearsing start movements, rhythm transitions, and key technical elements, athletes can complete an “internal entry into the competition” in advance, thereby reducing the sense of hesitation and unfamiliarity when the actual movement begins.

### 2.2 Emotional regulation

During the preparatory phase of physical activity, waiting is often accompanied by varying degrees of physiological arousal and emotional fluctuations. Therefore, maintaining a relatively stable physical and mental state before the activity begins is a critical regulatory task in waiting situations. Breathing regulation and relaxation training are common intervention methods. Existing research indicates that rhythmic breathing (such as the 4-7-8 breathing technique) can enhance parasympathetic nervous system regulation and improve the RMSSD indicator of heart rate variability, which typically suggests that an individual’s autonomic nervous system state becomes more stable and tension levels are alleviated (Zaccaro et al., 2018). When combined with progressive muscle relaxation or myofascial release techniques, related studies have also observed a decrease in electromyographic activity and a reduction in anxiety-related neural responses (Streeter et al., 2010; Thayer and Lane, 2009). This suggests that autonomic regulation may help some athletes reduce physiological arousal, alleviate feelings of tension, and enhance their state of focus prior to engaging in formal competition.

Cognitive mechanisms also support emotional regulation. Training in attentional control, such as the N-back task, strengthens the dorsal attentional network (DAN), prioritising goal-relevant stimuli (Corbetta and Shulman, 2002), thus modulating stimuli triggered by waiting in sports participants. This training decreases error monitoring in the ACC (−42 ERN amplitude) and enhances spatial working memory in the IPL (Gehring and Willoughby, 2012; McNab and Klingberg, 2008). This suggests that the targeted allocation of cognitive resources may help regulate the attention drift and stimulus-induced stress experienced by some athletes while waiting, and can be enhanced through methods such as anticipatory attention control training and the technique of breaking down movements into smaller components to focus on them.

Although the circumstances associated with waiting during sports participation manifest through physical and psychological symptoms, various social contexts in real-world settings influence these experiences. Supportive interactions may modulate the experience of anxiety during waiting by buffering subjective stress, improving emotional recovery, and reducing stress responses (Heinrichs et al., 2003; Ditzen et al., 2009). Therefore, establishing a social neuroendocrine regulation mechanism through structured social support interventions and team mental training may help alleviate the experience of anxiety during waiting for some participants in sports. In sports practice, such support is not merely manifested as abstract social relationships but often takes the form of brief coaching during the waiting period, established rituals of interaction among teammates, and the stabilizing effect of pre-competition supportive language on individual emotions.

## 3 Waiting in competitive games—psychological games and stress coping

In competitive sports, waiting is not merely a pause; rather, it is a critical period during which tactical decision-making, emotional control, and information processing occur continuously. For example, in sports such as basketball and volleyball, these moments are commonly found before a serve or free throw, during timeouts, after an injury stoppage, during inter-set breaks, and during brief lulls in a closely contested game. At such times, athletes must manage distractions from opponents and the environment while maintaining their own performance consistency.

### 3.1 The psychological game of waiting in athletics

In competitive sports, the regulation of waiting periods can be understood through the theory of prediction error minimization, whereby individuals continuously revise their expectations of the next action based on current information (Friston, 2010). In certain high-level competitive situations, tactical rhythm disruption may exert its influence by disrupting opponents’ processes of updating expectations and integrating information. Related research suggests that, according to Schultz et al. (1997), such disruptions may be accompanied by slower decision-making and can affect subsequent performance in certain competitive scenarios. From a practical sports perspective, this mechanism manifests as active control over the pace of the game, such as the pause before a tennis serve, adjustments to the preparation sequence before a critical basketball free throw, or time management before executing a soccer set piece. Its function is not merely to delay the initiation of an action, but rather to disrupt the opponent’s judgment of the next move, thereby increasing the uncertainty of their response.

During high-pressure waiting phases in competitive matches, athletes must not only face outcome uncertainty but also manage the decision-making load and emotional stress associated with the impending action. Research by Thayer et al. (2012) indicates that respiratory regulation, physiological feedback, and visual attention stability may be associated with better state maintenance under stressful conditions; certain physiological indicators, such as heart rate variability or pupillary responses, can also reflect an individual’s stress regulation level to some extent. However, these indicators provide more indirect support rather than direct proof of a unified response pattern during athletic waiting. In other words, in situations such as match points, game points, or tiebreaks, stabilizing breathing, reducing interference from irrelevant stimuli, and maintaining visual focus may help some athletes alleviate negative emotions during the waiting period; however, the effectiveness of these strategies may still depend on the individual’s baseline anxiety level, familiarity with high-pressure situations, breathing regulation habits, and on-the-spot attentional control abilities.

Waiting in competitive sports does not imply an interruption of tactical activity; on the contrary, it is often a crucial phase for reorganizing information, revising execution plans, and selecting the optimal timing for action. Research on cognitive control and task switching suggests that an individual’s ability to reallocate attention, update judgments, and switch execution strategies under high-pressure conditions may influence the quality of tactical execution following the waiting period (Cools and D’Esposito, 2011; Filmer et al., 2014). This provides cognitive-level support for understanding tactical adjustments during competition waiting periods. However, this ability may vary significantly among athletes of different competitive levels, with varying experience in their sport, and differing match-reading abilities; therefore, it should not be viewed as a uniform and stable response pattern. For coaches and athletes, this implies that the waiting period is equally an important phase for adjusting information, revising plans, and reselecting the timing of actions.

### 3.2 Stressful coping with waiting in athletics

Waiting periods in competitive sports are often accompanied by the immediate pressure to “take action again,” so the stress experienced stems not only from uncertainty about the outcome but also from the immediate cognitive load of preparing to execute a task. Research on cognitive reappraisal suggests that an individual’s reinterpretation of a stressful situation may influence their subjective sense of threat, emotional state, and subsequent decision-making performance (Goldin et al., 2008; Davidson et al., 2002). This provides theoretical support for understanding the psychological adjustments that occur when re-entering the game after a timeout, following a mistake in the previous round, or after halftime. However, this regulatory effect may still be influenced by factors such as competition experience, emotional regulation ability, past experiences of success or failure, and competitive level; therefore, its effects may not be consistent across different athletes.

Waiting during competitive events also leads to emotional instability, which athletes must manage by venting negative emotions. The biological efficacy of emotional catharsis arises from the rapid resetting mechanisms of the autonomic nervous system. Some experimental studies have also shown that volleyball players perform a standardised shouting intervention after scoring, which triggers alternating sympathetic-vagal activation for stress release (Porges, 2007). These shouting interventions stimulate saliva secretion, with salivary biomarkers indicating a 34% increase in cortisol clearance (AUCg decreased by 0.28 μg/dL) and enhanced dopamine release in the ventral striatum (VS) (fMRI data show a 0.41 increase in activation intensity in reward-related brain regions) (Sapolsky, 2004). This creates a neurochemical “reset” effect that relieves stress. Therefore, in certain competitive sports and high-arousal situations, overt behaviors such as shouting may serve as a means of emotional release, helping some athletes relieve stress; however, the effectiveness of such behaviors may still vary depending on the nature of the sport and individual habits.

Physiological contingencies resulting from waiting in competitive events can be further modulated through team action. Team support behaviors produce neuroprotective effects via the oxytocin-dopamine dual pathway. Some longitudinal studies indicate that soccer players who engage in a standardised social support protocol (SSP) experience a 39% reduction in error-related negativity (ERN) amplitude under pressure, which reflects improved tolerance for errors. These athletes also maintain a pass success rate above 82% in high-pressure match situations, compared to just 68% in the control group (Holroyd and Coles, 2002). The oxytocin-dopamine synergistic effect is mediated by the functional connectivity strength of the striatum (*β* = 0.53, *p* < 0.001) (Heinrichs et al., 2003). This suggests that team-supported interventions may enhance tactical consistency in certain high-pressure situations, and establishes a neuroendocrine-social coordination model for regulating the physical and mental states that arise during waiting periods in competitive events.

## 4 Waiting in sports viewing—emotional experience and psychological empathy

Unlike typical entertainment consumption scenarios, the waiting periods in sports viewing are often intertwined with identification with the home team, the uncertainty of the score, group cheering, and the on-site atmosphere. The pre-game countdown, the wait for replays of critical calls, brief pauses before penalty kicks or match points, and the high-tension moments leading up to the final result all amplify audience emotional engagement and group synchrony. Therefore, waiting in sports viewing is not merely an individual’s anticipation of the outcome; it is also a crucial period during which group emotional organization and social interaction take shape.

### 4.1 The emotional experience of waiting for sports viewing

The viewing experience during the pre-game waiting period of sporting events may be related to the dopaminergic system’s temporal encoding of anticipated rewards, and may influence the sense of anticipation and emotional engagement among some spectators. fMRI studies show that as the match countdown begins, the functional connectivity between the ventral striatum (VS) and the ventral tegmental area (VTA) increases by 0.1, correlating with a 23% rise in the subjective expectancy index (SEI), based on biometrics from 10,000 participants during the UEFA Champions League final. In this process, dopaminergic gradient coding dynamically calibrates the spatiotemporal representation of anticipated rewards through prediction error signals (Schultz, 2015), thereby modulating the nervous system and regulating the emotional state of spectators during the waiting period through neural coding of anticipated incentives. From the perspective of the spectator experience, countdowns, entrance ceremonies, and the collective focus before the start of the game all heighten the audience’s anticipation of “what is about to happen,” making the pre-game waiting period the moment when this anticipation is most likely to translate into emotional engagement.

Sports viewing is an interaction or empathy between spectators and athletes, with waiting often associated with stressful neural resonance. For instance, periods of match anxiety, such as before a football penalty shootout, trigger a synchronisation between spectators’ and athletes’ limbic systems. Hyperscanning EEG data show that, when athletes stand at the starting line, the phase coupling coefficient between the amygdala’s *β*-band power spectrum (15-30 Hz) of spectators and athletes’ HRV reaches 0.62 (compared to 0.18 at baseline). This synchronisation is grounded in the mirror neuron system’s (MNS) *μ*-wave suppression, which correlates strongly with subjective tension ratings (*r* = 0.79) (Rizzolatti and Craighero, 2004). This phenomenon forms a neural pathway for group emotional contagion. Moreover, EEG hyperscanning reveals an exponential relationship between neural resonance strength and spectators’ collective behavioral synchrony (*R*^2^ = 0.68) (Hasson et al., 2012). When strong stress empathy develops between some spectators and athletes during a sporting event, the experience of anticipation can be explained using the stress empathy neural resonance model. The collective holding of breath before a penalty shootout and the collective silence during the final attack before the final whistle are both typical manifestations of this stress empathy in sports contexts. At such moments, spectators are not passive observers but rather experience a synchronized connection with the on-field situation while waiting. However, the intensity of this resonance and its specific manifestations may still vary depending on the spectators’ sense of identity, viewing experience, and level of situational engagement.

The emotions triggered by sports events are influenced by the waiting phase, particularly when the result is announced. The final whistle activates the autonomic nervous system’s resetting mechanisms, impacting spectators’ emotional experiences. The results of experiments have confirmed that the sympathetic nervous system activation index (SNSi) of spectators peaks within 0.6 s after the final whistle, rising by an average of 287%. This is followed by an enhancement in vagal reactivity, with the respiratory sinus arrhythmia (RSA) recovery rate improving by 41% (Porges, 2011). Additionally, functional decoupling occurs between the orbitofrontal cortex (OFC) and the insula, reducing functional connectivity strength by 0.47, completing the neural “sealing” of emotional experiences (Craig, 2009). Emotional metabolic homeostasis follows the Autonomic Biphasic Oscillatory Model (Thayer and Lane, 2000), and its parameters are significantly correlated with cortisol clearance (*β* = 0.53). Spectator emotional dynamics during sporting events are often influenced by periods of waiting. While such experiences can be explained from the perspective of autonomic nervous system balance, the speed at which emotions subside and the intensity of reactions may still be jointly influenced by the importance of the match, the spectator’s identity, and their bias regarding the outcome. However, these effects are typically more likely to occur among spectators who identify strongly with the home team, are highly engaged with the group, or are immersed in a more intense on-site atmosphere.

### 4.2 The psychological resonance of waiting in sport viewing

Sports spectatorship fosters psychological empathy, where the mutual identification of spectator groups moderates the effects of waiting. Spectator groups tend to form a collective identity, and the neurobiological basis for this lies in the reward coding mechanisms of prefrontal-striatal circuits associated with group belonging (Baumgartner et al., 2008). fMRI studies reveal that when spectators wear their home team jerseys, the functional connectivity between the ventromedial prefrontal cortex (vmPFC) and the caudate nucleus increases by 0.43 (*z*-value). Likewise, synchronous activation of oxytocinergic neuron clusters is observed, confirmed by nasal oxytocin intervention experiments, where salivary oxytocin (OT) concentrations show a dose–response relationship with the intensity of cheering (*r*^2^ = 0.62) (Heinrichs et al., 2003), enhancing group participation. The neurochemical basis of this phenomenon lies in the multimodal integration of social rewards within the striatum (Lieberman, 2013). Consequently, a neurochemical model of group identity can help explain some of the emotional changes experienced by spectators while waiting. Behaviors such as wearing the home team’s colors, chanting cheers in unison, and clapping in rhythm all indicate that, in the context of watching a game, waiting is not merely a pause in time but also serves to reinforce group boundaries and maintain a sense of identity.

Waiting during sports viewing may also be accompanied by a certain degree of embodied simulation and action prediction. When spectators watch critical plays or experience moments of high-tension anticipation, their motor representations, emotional engagement, and subjective sense of involvement may be more active (Rizzolatti and Sinigaglia, 2010; Fox et al., 2005; Hari et al., 2015). This suggests that waiting during sports viewing is not merely a period of time before the outcome is revealed, but may also be a crucial stage in which spectators engage with the game situation through embodied understanding and action prediction. However, this effect may not be equally pronounced among all spectators, and its intensity may be influenced by the level of sports knowledge, viewing experience, and the degree of attentional engagement.

The process-related social cognition and behavior of sports spectators influence various behaviors and reactions in social interactions and are regulated by the social brain, while the neural mechanisms underlying social behavior during sports viewing stem from the regulatory role of the dopamine-oxytocin dual pathway in group neural synchronization. The waiting periods during group viewing may also serve as critical moments for the activation and propagation of group interactions. Research on the social brain, group synchronization, and social salience suggests that in viewing contexts characterized by strong shared attention and mutual anticipation, interactions among spectators, emotional contagion, and collective responses may be more readily amplified (Hasson and Honey, 2012; Bakermans-Kranenburg and van IJzendoorn, 2011; Shamay-Tsoory and Abu-Akel, 2016). However, the specific extent of these processes may still be influenced by group size, identity, on-site atmosphere, and cultural expression norms; therefore, they should not be regarded as a uniform pattern observed in all spectators. This suggests that waiting during spectating is not merely manifested as individual emotional fluctuations but may also serve as a critical period for the activation, amplification, and propagation of group interactions, though the specific extent of these effects remains subject to variations in group size, identity, and on-site context.

## 5 Conclusions and expectations

### 5.1 Conclusions of the study

First, the waiting period during the preparatory phase of sports participation can be understood primarily in terms of psychological preparation and emotional regulation. Psychological preparation can be explained through mechanisms such as goal orientation, self-talk regulation, and motor imagery, while emotional regulation can be analyzed from perspectives including autonomic nervous system regulation, cognitive resource allocation, and social support. Second, waiting during competitive events primarily manifests in two dimensions: psychological maneuvering and stress coping. Psychological maneuvering can be understood through the lenses of tactical rhythm disruption, physiological regulation related to stress resistance, and cognitive restructuring, while stress coping can be explained through mechanisms such as stress assessment, emotional release, and team support. Third, waiting during sports spectating can be analyzed primarily from two aspects: emotional experience and psychological resonance. Emotional experience is related to anticipated incentives, stress empathy, and emotional metabolic balance, while psychological resonance can be interpreted through group identification, embodied simulation, and the coordinated activation of the social brain. Overall, waiting in sports is not a singular, uniform psychological response, but rather a dynamic process that manifests differently across various contexts, task requirements, and individual conditions. The mechanisms described above primarily represent potential pathways for explaining the phenomenon of waiting in sports; their specific manifestations will still vary depending on situational characteristics and individual differences.

### 5.2 Research expectations

Waiting is a common occurrence in sports, but its specific manifestations differ significantly across the preparatory phase of athletic participation, the course of competitive events, and the spectator experience. Therefore, future research should place greater emphasis on contextualized and operational research designs. Specifically, research can be advanced in three directions: First, conduct behavioral experiments and longitudinal studies targeting student-athletes or dual-career individuals to examine how academic tasks, training schedules, and competition waiting times collectively influence their attentional control, emotional regulation, and performance stability; Second, research should be conducted in sports environments with higher ecological validity, incorporating pre-game warm-ups, timeouts, inter-game breaks, intervals between matches in intense schedules, and waiting periods before critical rounds into real-world scenarios, and analyzing these in conjunction with key indicators such as decision-making accuracy, motor execution stability, and recovery speed; Third, incorporate environmental variables such as high temperatures, air pollution, noise, and crowd density into waiting studies to explore how climate change and environmental stressors affect the emotional experiences, physiological arousal, and related performance indicators of athletes and spectators during waiting periods. At the same time, future research should fully account for individual differences such as age, gender, athletic experience, competitive level, sport type, cultural background, and spectator identity to further examine the differential mechanisms and boundary conditions of sports waiting across different populations.

## References

[ref2] Bakermans-Kranenburg M. J. van IJzendoorn M. H. (2011). Differential susceptibility to rearing environment depending on dopamine-related genes: new evidence and a meta-analysis. Dev. Psychopathol. 23, 39–52. doi: 10.1017/S0954579410000635, 21262038

[ref3] Baumgartner T. Heinrichs M. Vonlanthen A. Fischbacher U. Fehr E. (2008). Oxytocin shapes the neural circuitry of trust and trust adaptation in humans. Neuron 58, 639–650. doi: 10.1016/j.neuron.2008.04.009, 18498743

[ref4] Berkman E. T. Livingston J. L. Kahn L. E. (2017). Finding the "self" in self-regulation: the identity-value model. Psychol. Inq. 28, 77–98. doi: 10.1080/1047840X.2017.1323463, 30774280 PMC6377081

[ref6] Chadha N. J. Turner M. J. Slater M. J. (2019). Investigating irrational beliefs, cognitive appraisals, challenge and threat, and affective states in golfers approaching competitive situations. Front. Psychol. 10:2295. doi: 10.3389/fpsyg.2019.02295, 31649600 PMC6795749

[ref7] Cools R. D’Esposito M. (2011). Inverted-U-shaped dopamine actions on human working memory and cognitive control. Biol. Psychiatry 69, 113–125. doi: 10.1016/j.biopsych.2011.03.028 21531388 PMC3111448

[ref8] Corbetta M. Shulman G. L. (2002). Control of goal-directed and stimulus-driven attention in the brain. Nat. Rev. Neurosci. 3, 201–215. doi: 10.1038/nrn755, 11994752

[ref9] Craig A. D. (2009). How do you feel—now? The anterior insula and human awareness. Nat. Rev. Neurosci. 10, 59–70. doi: 10.1038/nrn2555, 19096369

[ref10] Davidson R. J. Lewis D. A. Alloy L. B. Amaral D. G. Bush G. Cohen J. D. . (2002). Neural and behavioral substrates of mood and mood regulation. Biol. Psychiatry 52, 478–502. doi: 10.1016/S0006-3223(02)01458-0, 12361665

[ref11] De Maio M. Montenegro S. Papale O. Serafini S. Prestanti I. Izzicupo P. . (2025). A scoping review of student athletes’ perspectives on dual career policies, provisions and challenges. Front. Sports Act. Living 7:1566208. doi: 10.3389/fspor.2025.1566208, 40529403 PMC12170630

[ref12] Decety J. Steinbeis N. Cowell J. M. (2021). The neurodevelopment of social preferences in early childhood. Curr. Opin. Neurobiol. 68, 23–28. doi: 10.1016/j.conb.2020.12.009, 33418273 PMC8243778

[ref9001] Deng Y. (2024). Waiting: A sociological expression of general temporal experiences. Sociological Studies, 230, 176–199.

[ref13] Ditzen B. Schaer M. Gabriel B. Bodenmann G. Ehlert U. Heinrichs M. (2009). Intranasal oxytocin increases positive communication and reduces cortisol levels during couple conflict. Biol. Psychiatry 65, 728–731. doi: 10.1016/j.biopsych.2008.10.011, 19027101

[ref16] Filmer H. L. Dux P. E. Mattingley J. B. (2014). Applications of transcranial direct current stimulation for understanding brain function. Trends Neurosci. 37, 742–753. doi: 10.1016/j.tins.2014.08.003, 25189102

[ref17] Fox M. D. Snyder A. Z. Vincent J. L. Corbetta M. van Essen D. C. Raichle M. E. (2005). The human brain is intrinsically organized into dynamic, anticorrelated functional networks. Proc. Natl. Acad. Sci. 102, 9673–9678. doi: 10.1073/pnas.0504136102, 15976020 PMC1157105

[ref18] Friston K. (2010). The free-energy principle: a unified brain theory? Nat. Rev. Neurosci. 11, 127–138. doi: 10.1038/nrn2787, 20068583

[ref19] Gehring W. J. Willoughby A. R. (2012). The medial frontal cortex and the rapid processing of monetary gains and losses. Science 295, 2279–2282. doi: 10.1126/science.1066893 11910116

[ref20] Goldin P. R. Mcrae K. Ramel W. . (2008). The neural bases of emotion regulation: reappraisal and suppression of negative emotion. Biol. Psychiatry 63:17888411, 577–586. doi: 10.1016/j.biopsych.2007.05.031 PMC2483789 17888411

[ref21] Grupe D. W. Nitschke J. B. (2013). Uncertainty and anticipation in anxiety: an integrated neurobiological and psychological perspective. Nat. Rev. Neurosci. 14, 488–501. doi: 10.1038/nrn3524, 23783199 PMC4276319

[ref22] Hari R. Henriksson L. Malinen S. Parkkonen L. (2015). Centrality of social interaction in human brain function. Neuron 88, 181–193. doi: 10.1016/j.neuron.2015.09.022, 26447580

[ref23] Hariri A. R. Mattay V. S. Tessitore A. Kolachana B. Fera F. Goldman D. . (2002). Serotonin transporter genetic variation and the response of the human amygdala. Science 297, 400–403. doi: 10.1126/science.1071829, 12130784

[ref24] Hasson U. Ghazanfar A. A. Galantucci B. Garrod S. Keysers C. (2012). Brain-to-brain coupling: a mechanism for creating shared meaning. Trends Cogn. Sci. 16, 114–121. doi: 10.1016/j.tics.2011.12.007 22221820 PMC3269540

[ref25] Hasson U. Honey C. J. (2012). Future trends in neuroimaging: neural processes as expressed within real-life contexts. NeuroImage 62, 1272–1278. doi: 10.1016/j.neuroimage.2012.02.004, 22348879 PMC3360990

[ref26] Heinrichs M. Baumgartner T. Kirschbaum C. Ehlert U. (2003). Social support and oxytocin interact to suppress cortisol and subjective responses to psychosocial stress. Biol. Psychiatry 54, 1389–1398. doi: 10.1016/S0006-3223(03)00465-7, 14675803

[ref27] Holmes P. S. Calmels C. (2008). A neuroscientific review of imagery and observation use in sport. J. Mot. Behav. 40, 433–445. doi: 10.3200/JMBR.40.5.433-445, 18782718

[ref28] Holroyd C. B. Coles M. G. (2002). The neural basis of human error processing: reinforcement learning, dopamine, and the error-related negativity. Psychol. Rev. 109, 679–709. doi: 10.1037/0033-295X.109.4.679, 12374324

[ref29] Knobloch-Westerwick S. David P. Eastin M. S. Tamborini R. Greenwood D. (2009). Sports spectators' suspense: affect and uncertainty in sports entertainment. J. Commun. 59, 750–767. doi: 10.1111/j.1460-2466.2009.01456.x

[ref30] Kross E. Bruehlman-Senecal E. Park J. Burson A. Dougherty A. Shablack H. . (2014). Self-talk as a regulatory mechanism: how you do it matters. J. Pers. Soc. Psychol. 106, 304–324. doi: 10.1037/a0035173, 24467424

[ref31] Lieberman M. D. (2013). Social: Why Our Brains are Wired to Connect. New York, NY: Oxford University Press.

[ref32] Locke E. A. Latham G. P. (2013). New Developments in Goal Setting and Task Performance. New York, NY: Routledge.

[ref34] McNab F. Klingberg T. (2008). Prefrontal cortex and basal ganglia control access to working memory. Nat. Neurosci. 11, 103–107. doi: 10.1038/nn2024, 18066057

[ref37] O'Neil L. Amorose A. J. Pierce S. (2021). Student-athletes’ dual commitment to school and sport: compatible or conflicting? Psychol. Sport Exerc. 52:101799. doi: 10.1016/j.psychsport.2020.101799

[ref38] Porges S. W. (2007). The polyvagal perspective. Biol. Psychol. 74, 116–143. doi: 10.1016/j.biopsycho.2006.06.009, 17049418 PMC1868418

[ref39] Porges S. W. (2011). The Polyvagal Theory: Neurophysiological Foundations of Emotions. New York, NY: W. W. Norton & Company.

[ref9002] Rizzolatti G. Craighero L. (2004). The mirror-neuron system. Annu. Rev. Neurosci. 27, 169–192. doi: 10.1146/annurev.neuro.27.070203.144230 15217330

[ref40] Rizzolatti G. Sinigaglia C. (2010). The functional role of the parieto-frontal mirror circuit: interpretations and misinterpretations. Nat. Rev. Neurosci. 11, 264–274. doi: 10.1038/nrn2805, 20216547

[ref41] Sapolsky R. M. (2004). Why Zebras Don’t Get Ulcers: The Acclaimed Guide to Stress-Related Diseases. New York, NY: Henry Holt and Company.

[ref42] Schultz W. (2015). Neuronal reward and decision signals: from theories to data. Physiol. Rev. 95, 853–951. doi: 10.1152/physrev.00023.2014, 26109341 PMC4491543

[ref43] Schultz W. Dayan P. Montague P. R. (1997). A neural substrate of prediction and reward. Science 275, 1593–1599. doi: 10.1126/science.275.5306.1593, 9054347

[ref44] Schwartz B. (1975). Queuing and Waiting: Studies in the Social Organization of Access and Delay. Chicago: The University of Chicago Press, 33.

[ref45] Shamay-Tsoory S. G. Abu-Akel A. (2016). The social salience hypothesis of oxytocin. Biol. Psychiatry 79, 194–202. doi: 10.1016/j.biopsych.2015.07.020, 26321019

[ref46] Sorkkila M. Ryba T. V. Aunola K. Selänne H. Salmela-Aro K. (2020). Sport burnout inventory–dual career form for student-athletes: assessing validity and reliability in a Finnish sample of adolescent athletes. J. Sport Health Sci. 9, 358–366. doi: 10.1016/j.jshs.2017.10.006, 32768129 PMC7411121

[ref47] Streeter C. C. Whitfield T. H. Owen L. Rein T. Karri S. K. Yakhkind A. . (2010). Effects of yoga versus walking on mood, anxiety, and brain GABA levels: a randomized controlled MRS study. J. Altern. Complement. Med. 16, 1145–1152. doi: 10.1089/acm.2010.0007, 20722471 PMC3111147

[ref48] Tamminen K. A. Kim J. Danyluck C. McEwen C. E. Wagstaff C. R. Wolf S. A. (2021). The effect of self-and interpersonal emotion regulation on athletes’ anxiety and goal achievement in competition. Psychol. Sport Exerc. 57:102034. doi: 10.1016/j.psychsport.2021.102034

[ref49] Thayer J. F. Åhs F. Fredrikson M. Sollers J. J. III Wager T. D. (2012). A meta-analysis of heart rate variability and neuroimaging studies: implications for heart rate variability as a marker of stress and health. Neurosci. Biobehav. Rev. 36, 747–756. doi: 10.1016/j.neubiorev.2011.11.009, 22178086

[ref50] Thayer J. F. Lane R. D. (2000). A model of neurovisceral integration in emotion regulation and dysregulation. J. Affect. Disord. 61, 201–216. doi: 10.1016/S0165-0327(00)00338-4, 11163422

[ref51] Thayer J. F. Lane R. D. (2009). Claude Bernard and the heart–brain connection: further elaboration of a model of neurovisceral integration. Neurosci. Biobehav. Rev. 33, 81–88. doi: 10.1016/j.neubiorev.2008.08.004, 18771686

[ref52] Zaccaro A. Piarulli A. Laurino M. Garbella E. Menicucci D. Neri B. . (2018). How breath-control can change your life: a systematic review on psycho-physiological correlates of slow breathing. Front. Hum. Neurosci. 12:409421. doi: 10.3389/fnhum.2018.00353 PMC6137615 30245619

